# The Importance of Mind–Body in Pilates Method in Patients with Chronic Non-Specific Low Back Pain—A Randomized Controlled Trial

**DOI:** 10.3390/jcm13164731

**Published:** 2024-08-12

**Authors:** Pablo Vera-Saura, Jesús Agudo-Pavón, Dara María Velázquez-Torres, María Martín-Alemán, Felipe León-Morillas, Aday Infante-Guedes, David Cruz-Díaz

**Affiliations:** 1 Department of Health Sciences, Faculty of Health Sciences, University of Jaén, 23071 Jaén, Spain; pablo.vera.saura@hotmail.com (P.V.-S.); jesusagudop@gmail.com (J.A.-P.); dcruz@ujaen.es (D.C.-D.); 2 Grupo ICOT Arnao (Gran Canaria), 35200 Telde, Spain; supervision.arnao@grupoicot.es (D.M.V.-T.); arnao@grupoicot.es (M.M.-A.); 3 Department of Physiotherapy, Faculty of Physiotherapy, Podiatry and Therapy Occupational, Catholic University of Murcia (UCAM), 30107 Murcia, Spain; 4 Faculty of Health Sciences, University of Atlántico Medio, 35017 Las Palmas de Gran Canaria, Spain; aday.infante@pdi.atlanticomedio.es

**Keywords:** chronic non-specific low back pain, Pilates, mind–body connection, functional disability, kinesiophobia

## Abstract

**Background/Objectives:** Chronic non-specific low back pain (CNSLBP) is a prevalent condition causing significant distress and healthcare costs globally. Despite various treatments, effective management remains challenging. Pilates, recognized for its focus on core strength and postural alignment, has emerged as a promising intervention. This study investigates the impact of mind–body in Pilates for directing participants on CNSLBP outcomes. **Methods:** A randomized controlled trial was conducted with 67 participants, aged 18 to 65 years, suffering from CNSLBP. They were allocated into two groups: Pilates with mind–body cueing (*n* = 34) and Pilates without cueing (*n* = 33). Both groups underwent 60 min sessions twice weekly for 8 weeks. Outcome measures included pain intensity (Visual Analogue Scale), functional disability (Roland Morris Disability Questionnaire), fear of movement (Tampa Scale of Kinesiophobia), and adherence (percentage of sessions attended). Statistical significance was determined through repeated measures ANOVA. **Results:** Both groups showed significant improvement in pain reduction, functional ability, and kinesiophobia. However, the mind–body group demonstrated a statistically significant reduction in kinesiophobia compared to the non-cueing group (*p* = 0.048), indicating the potential additional benefit of mind–body cueing in managing movement-related fear in CNSLBP. **Conclusions:** This study underscores the effectiveness of an 8-week Pilates intervention in managing CNSLBP, highlighting the added value of mind–body cueing in reducing fear of movement. These findings suggest incorporating mind–body cueing in Pilates could enhance the therapeutic benefits, particularly for patients with high levels of movement-related fear, potentially improving long-term adherence to physical activity and rehabilitation outcomes.

## 1. Introduction

Chronic non-specific low back pain (CNSLBP) is a pervasive musculoskeletal ailment that exerts a substantial burden on individuals and healthcare systems worldwide [1]. Defined as pain and discomfort in the region between the ribcage and gluteal folds, CNSLBP lacks a specific structural or pathological cause, making its management a complex challenge [2]. Epidemiological data reveal that CNSLBP represents a significant public health issue, affecting up to 80% of the global population at some point in their lives [3]. The economic implications are profound, with substantial healthcare expenditures associated with its treatment, disability compensation, and lost productivity [4].

In recent years, exercise-based interventions have emerged as a cornerstone in the management of CNSLBP, with Pilates exercise gaining prominence as an effective and holistic approach [5]. Pilates, developed by Joseph Pilates in the early 20th century, is a mind–body exercise system that focuses on core stability, flexibility, and overall body strength through controlled and precise movements [6]. Its emphasis on postural alignment, muscle endurance, and mindful movement makes it a promising intervention for individuals suffering from CNSLBP. An intriguing aspect of Pilates instruction that has garnered increasing attention is the concept of “cueing”. Cueing refers to the verbal and tactile guidance provided by instructors to enhance the execution of specific exercises, ensuring proper alignment and muscle engagement [7]. While cueing has been an integral part of Pilates instruction, its specific role and potential impact on outcomes related to CNSLBP are not yet fully understood.

Mind–body cueing in Pilates serves several critical functions that make it a vital component of the practice, particularly in the context of managing CNSLBP:

○ Enhanced Mind–Body Connection: Mind–body cueing promotes a heightened awareness of body alignment, posture, and movement. For individuals with CNSLBP, this heightened awareness can be crucial in reducing pain and preventing injury by facilitating better body mechanics during exercises [8].
○ Precise Muscle Activation: Instructors use cues to direct attention to specific muscles that need to be engaged during exercises. This ensures that the targeted muscle groups are activated effectively, aiding in the development of core strength and stability—a fundamental aspect of CNSLBP management [9].
○ Safety and Injury Prevention: Proper alignment and technique are paramount in Pilates, and mind–body cueing plays a pivotal role in ensuring participants perform movements safely. Individuals with CNSLBP are often cautious about exacerbating their pain, and mind–body cueing provides reassurance and guidance in executing exercises safely [10].
○ Motivation and Adherence: Effective cueing can enhance motivation and adherence to Pilates programs. By providing real-time feedback and encouragement, instructors can keep participants engaged and committed to their exercise routines, which is essential for long-term pain management [11].

Considering the complex interplay between mind–body cueing and its potential to influence outcomes in chronic non-specific low back pain (CNSLBP), it is essential to further investigate the specific mechanisms and benefits of this approach within Pilates-based interventions. This article seeks to comprehensively examine the potential impact of mind–body cueing in Pilates on CNSLBP-related outcomes. We hypothesize that incorporating mind–body cueing into Pilates exercise programs could lead to superior results compared to interventions that do not utilize this method.

## 2. Materials and Methods

### 2.1. Study Design

A double-blinded randomized controlled trial was conducted to assess the efficacy of Pilates with mind–body cueing compared to Pilates without cueing in individuals with chronic non-specific low back pain (CNSLBP). The study employed a parallel-group design and spanned 8 weeks, with two exercise sessions per week for both groups. The study received ethical approval from the Research Ethics Committee of the University of Jaén 1 November 2023 and was registered in the online database Clinicatrail.gov, managed by the National Library of Medicine of the United States, under registration number NCT06340191. Informed consent was obtained from all participants, and strict data confidentiality measures were implemented throughout the study. The number of participants required for the study was determined using the GPower 3.0.10 software. This calculation aimed to identify a 2.5-point difference in the RMQ, recognizing this as the minimal clinically important difference (MCID) for patients with chronic low back pain, which typically varies by about 10 points. The assumptions for this calculation included a standard deviation of 2.5 points, employing a two-tailed test, setting the alpha (α) level at 0.05, and aiming for an 80% power (beta). Based on these parameters, the ideal sample size was established as 32 individuals for each group.

### 2.2. Participants

Participants were recruited through multiple channels to ensure a diverse sample. This included advertisements posted in local healthcare facilities such as clinics and hospitals, as well as outreach through online platforms including social media, local community forums, and the university’s website. Additionally, we collaborated with local general practitioners and physiotherapists who referred eligible patients to the study. All potential participants were pre-screened via a telephone interview to confirm they met the inclusion criteria before being invited for a baseline assessment.

Inclusion criteria comprised individuals aged 18 to 65 years experiencing chronic non-specific low back pain (CNSLBP) for a minimum of 3 months. Participants underwent a thorough diagnostic process to confirm CNSLBP. Initial diagnosis was made by primary care physicians or specialists based on clinical history and physical examination. To further confirm CNSLBP, our research team conducted additional assessments including a comprehensive musculoskeletal examination and standardized diagnostic criteria. This included evaluation of pain characteristics, functional limitations, and exclusion of specific pathologies (e.g., herniated discs, spinal stenosis) through imaging studies such as MRI or X-rays when necessary. Only those who met the criteria for CNSLBP, characterized by pain without a specific structural cause, were included in the study.

Additionally, participants underwent a rigorous differential diagnosis process to exclude other potential causes of low back pain. This included a thorough clinical assessment for red flags such as unexplained weight loss, fever, history of cancer, recent trauma, and neurological deficits (e.g., cauda equina syndrome). Subjects presenting any of these alarm bells were excluded from the study to ensure the safety and appropriateness of the intervention. Furthermore, we performed screening for psychological factors, ensuring that participants did not have severe psychiatric conditions that could confound the outcomes of the study. Detailed patient history was collected, including lifestyle habits (e.g., smoking, alcohol consumption, physical activity levels), previous treatments (e.g., physiotherapy, medications, surgery), and other comorbidities (e.g., diabetes, hypertension, obesity). Participants also reported their typical clinical setting, which included primary care, specialist clinics, or physiotherapy centers. This comprehensive history helped to ensure a thorough understanding of each participant’s background and the factors potentially influencing their response to the intervention [12].

**Figure 1:**
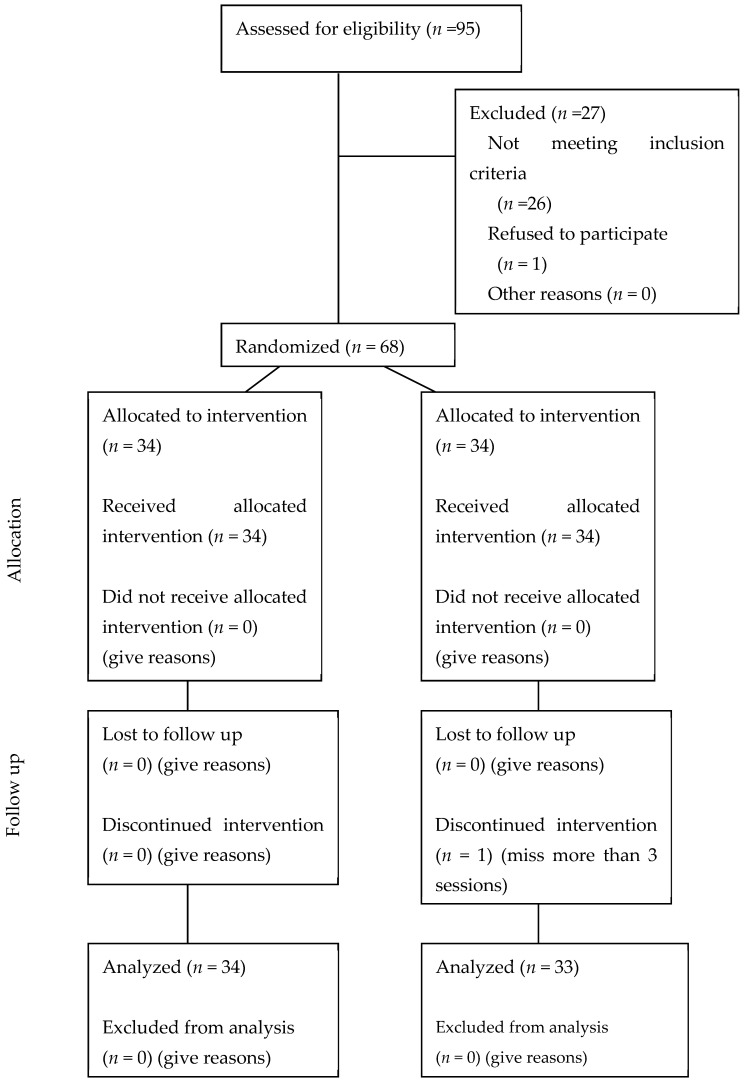
Flow chart of the study design and participant follow-up through the trial.

Randomization was performed using computer-generated random numbers generated by a biostatistician not involved in participant recruitment or intervention. Allocation concealment was ensured through the use of sequentially numbered, sealed envelopes. Participants were randomized in a 1:1 ratio into either Pilates with mind–body cueing (PMB) or Pilates alone without cueing (PA). Out of a total of 91 patients who responded to the clinical trial advertisement, 68 met the inclusion criteria, agreed to participate in the study, and were enrolled and randomized into the Pilates and mind–body cueing (PMB) intervention group or the control group, which practiced Pilates alone (PA) (Figure 1). The characteristics of both samples were similar at baseline (Table 1).

### 2.3. Intervention

Both groups participated in 60 min Pilates sessions, twice weekly, over 8 weeks. Certified Pilates instructors with extensive experience supervised all sessions. The Pilates program included a combination of mat-based and props-based exercises. Both groups were advised to maintain their usual activity and participants were excluded if they missed 3 or more training sessions.

**Figure 2:**
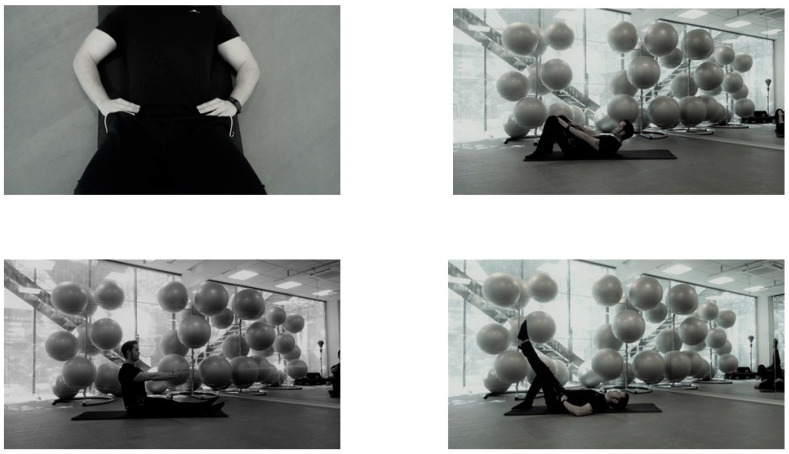
Pilates with mind–body cueing.

Intervention group (Pilates with mind–body cueing): Participants in this group received previous training (two small group sessions) focusing on explicit verbal and tactile cueing instructions during the execution of the exercises. Pilates exercises with mind–body cueing are designed to enhance participants’ awareness and implementation of fundamental Pilates principles. During this specific protocol, the intervention concentrated on developing the ability to distinguish and control the contraction of the deep abdominal muscles and the pelvic floor during the performance of the exercises. This includes focusing on correct breathing techniques and the maintenance of appropriate posture. Moreover, considerable emphasis was placed on the importance of adhering to core postural principles, such as lumbopelvic stability and intersegmental joint dissociation (Figure 2). These principles are crucial not only during the exercises, but also in incorporating them into daily activities to improve overall movement and posture. Additionally, intervention aims to provide evidence of the importance of the mind–body connection, which, although inherent to the Pilates method, may require specific training to fully integrate into practice. This highlights the need for deliberate and focused instruction to harness the full potential of this connection, enhancing both the effectiveness and the holistic benefits of Pilates.

Control group (Pilates alone without cueing): Participants in this group followed the same Pilates program as the intervention group but without the additional cueing instructions training. The protocol, described in detail in Table 2, was conducted by an expert Pilates physiotherapist instructor with 10 years of experience. The Pilates intervention program specifically focused on a series of targeted exercises aimed at enhancing lumbo-pelvic control. This included specific movements for hip disassociation and exercises designed to engage and stabilize the scapular girdle. Additionally, the program incorporated comprehensive articular work, emphasizing the improvement of mobility across the cervical, thoracic, and lumbar regions of the spine. These exercises were carefully structured to promote better alignment, flexibility, and overall spinal health.

### 2.4. Outcome Variables

Outcome variables included low back pain intensity (assessed by the Visual Analogue Scale), lumbar functionality (measured using the Oswestry Disability Index), and fear avoidance beliefs (evaluated with the Tampa Scale of Kinesiophobia), Measurements were taken at baseline and the conclusion of the 8-week intervention.

Roland Morris Disability Questionnaire (RMDQ): The RMDQ is a self-reported measure widely used to assess physical disability due to lower back pain. It consists of 24 items that reflect the range of activities that might be affected by back pain. The scoring is simple, with higher scores indicating greater disability. This questionnaire is known for its reliability and validity in measuring disability levels in individuals with back pain [13].

Tampa Scale of Kinesiophobia (TSK): The TSK is a 17-item scale used to measure fear of movement/(re)injury, which is a significant factor in chronic pain conditions, especially back pain. Each item is rated on a 4-point Likert scale, with total scores ranging from 17 to 68; higher scores represent greater kinesiophobia. The TSK has been validated in several studies and is crucial for understanding the role of fear avoidance in chronic pain [14].

Visual Analogue Scale (VAS): The VAS is a measurement instrument used for assessing the intensity of pain. It is a simple scale, typically a 10 cm line, where one end signifies “no pain” and the other end is “worst pain imaginable”. Patients mark on the line the point that they feel represents their perception of their current state. The VAS is highly regarded for its simplicity and has been widely used in various clinical settings to measure pain intensity [15].

Adherence to intervention: Adherence was monitored by tracking attendance to the Pilates sessions and was calculated as the percentage of sessions attended out of the total 16 sessions.

### 2.5. Statistical Analysis

A repeated measures analysis of variance (ANOVA) was employed to compare differences between groups in the outcome variables over time. Statistical significance was set at *p* < 0.05. Additionally, subgroup analyses were conducted to assess potential moderators and the effect of adherence to the intervention. A comprehensive statistical analysis was conducted to evaluate the impact of demographic variables on the outcomes of pain reduction, disability improvement, and kinesiophobia reduction. For continuous variables such as age, height, and weight, linear regression models were employed to assess their correlations with the outcomes. The regression analysis provided estimates of the slope, intercept, r-value, *p*-value, and standard error for each predictor–outcome pair. For categorical variables such as gender, education level, marital status, and occupation, an analysis of variance (ANOVA) was performed to determine if there were significant differences in the outcomes across the different groups.

## 3. Results

From the total sample of 68 participants, 34 patients completed the intervention in the PMB Group, and 33 in the PA Group, as one participant from this group was excluded for missing more than three training sessions. Adherence to the Pilates sessions was high, with an average attendance rate of 94.5% in the Pilates with mind–body cueing group and 92.8% in the Pilates without cueing group. No significant differences in adherence rates were observed between the two groups (*p* = 0.432).

Statistically significant improvements were observed in the study variables for both groups following the Pilates interventions, with and without the incorporation of specific mind–body cueing. For the Visual Analogue Scale (VAS), the PMB Group exhibited a reduction in pain from a mean of 5.2 (SD = 1.9) pre-intervention to 2.8 (SD = 1.7) post-intervention, while the PA Group showed a change from 4.9 (SD = 2.1) to 3.2 (SD = 1.9). In the Roland Morris Disability Questionnaire (RMDQ), the MBP Group demonstrated a decrease from 8.2 (SD = 2.9) to 3.1 (SD = 2.6), compared to the PA Group, which moved from 8.9 (SD = 2.6) to 4.3 (SD = 3.0). Regarding the Tampa Scale for Kinesiophobia (TSK), the MBP Group improved from 42.3 (SD = 6.5) to 34.6 (SD = 7.4), in contrast to the PA Group, which shifted from 41.2 (SD = 8.1) to 38.3 (SD = 7.5).

Statistical analysis revealed no significant differences between the groups in pain perception (VAS) and disability (RMDQ) post-intervention, with *p*-values of 0.372 and 0.088, respectively. However, for kinesiophobia (TSK), significant differences were found (*p* = 0.048), indicating a more pronounced benefit in the cueing group. The effect sizes for the VAS were moderate in both groups (Cohen’s d of 0.47 for the MBP Group and 0.49 for the PA Group). For the RMDQ, the effect sizes were small (0.17 for the MBP Group and 0.35 for the PA Group), suggesting modest improvements in pain-related disability. Notably, in the TSK, both groups exhibited large effect sizes (1.09 for the MBP Group and 1.12 for the PA Group), indicating significant improvements in reducing kinesiophobia. These results suggest that while Pilates intervention is generally effective for pain management and disability, the inclusion of specific mind–body cueing may have an additional notable impact on reducing movement-related fear in patients with chronic pain (Table 3).

The statistical analysis revealed that gender significantly influenced both pain reduction (*p* = 0.021) and kinesiophobia reduction (*p* = 0.043), indicating variations in outcomes between males and females. However, no significant correlations were found between age, height, or weight and the outcomes of pain reduction, disability improvement, or kinesiophobia reduction. Specifically, for pain reduction, the *p*-values for age (*p* = 0.856), height (*p* = 0.484), and weight (*p* = 0.988) indicated no significant impact. For disability improvement, the *p*-values for age (*p* = 0.515), height (*p* = 0.276), and weight (*p* = 0.647) were also not significant. Similarly, for kinesiophobia reduction, the *p*-values for age (*p* = 0.634), height (*p* = 0.531), and weight (*p* = 0.734) showed no significant correlations (Table 4).

For categorical variables, ANOVA results showed that education level, marital status, and occupation did not significantly affect the outcomes. Specifically, for pain reduction, the *p*-values were 0.396 for education, 0.457 for marital status, and 0.250 for occupation. For disability improvement, the *p*-values were 0.091 for gender, 0.282 for education, 0.714 for marital status, and 0.394 for occupation. For kinesiophobia reduction, the *p*-values were 0.657 for education, 0.384 for marital status, and 0.759 for occupation (Table 4).

## 4. Discussion

This study investigated the effect of incorporating previous mind–body cueing sessions before an 8-week Pilates mat-based program on pain, disability, and kinesiophobia in people with CNSLBP. Our results suggested that both interventions were effective in reducing pain, improving disability scores, and reducing kinesiophobia. However, we found no significant differences between the two groups regarding pain and disability. The main findings pertained to kinesiophobia, where there were statistical differences between the mind–body cueing group and the non-cueing group, suggesting its potential importance for patients with higher levels of pain catastrophizing or fear of movement.

Pilates principles employ a mind–body approach emphasizing breathing techniques, postural awareness, and specific muscle activity [10]. Incorporating breathing exercises into workouts is a simple, cost-free, and safe method that may enhance clinical outcomes across various dimensions [16]. Specific breathing techniques may lead to structural and/or functional changes in the brain, as evidenced by imaging studies [17]. These principles also include motor control and stabilization exercises, promoting activation of local stabilizer muscles such as the transversus abdominus or pelvic floor while moving the upper and lower extremities [18]. They play a crucial role in maintaining functional stability in the lumbopelvic region during daily tasks. Previous studies have shown that real-time US as a feedback-guided aid in transversus abdominis contraction is not superior to verbal or tactile feedback during an exercise program [19,20]. Strengthening the lumbopelvic muscles helps maintain spinal stability during movements and enhances neuromuscular recruitment patterns, reducing pain levels [21]. These results align with our findings, where both groups improved in pain and disability with tactile and verbal cues, even though more specific mind–body cueing at the beginning was not superior. In our study, we only provided information about specific transversus abdominus contractions in the mind–body cueing group. However, the impact of applying Pilates principles on pain and disability management remains uncertain [22,23].

Pilates principles can be taught directly during exercise sessions or reviewed in introductory sessions where participants learn and practice key principles [24]. However, there is no standardized procedure, which may impact outcomes in pain, disability, and kinesiophobia. This study is the first to investigate the role of providing initial, specific cueing and information versus incorporating it during the general Pilates exercise program. Our investigation hypothesized that integrating introductory sessions could improve patient outcomes in pain reduction, disability management, and kinesiophobia by enhancing proprioceptive input, neuromuscular control, and motor learning, while reducing hypervigilance. This mind–body approach in the introductory lessons implies specific motor control exercises before starting a general Pilates mat-based program. Xu et al. [25] showed that motor control exercises could reconfigure brain mapping, counteract adverse brain alterations, promote exercise-induced pain reduction, facilitate anti-inflammatory reactions, maintain typical neural activation, and enhance morphological deficiencies in patients with NSLBP.

Pain and disability are core outcomes in clinical trials in non-specific low back pain research [26]. A recent systematic review with meta-analysis found that at least 1 or 2 sessions per week of Pilates, over 3 to 9 weeks, could be more effective than other exercise interventions for managing pain and disability in people with NSLBP [27]. In our study, both groups showed significant improvement after 8 weeks of treatment. Our results align with previous studies regardless of when mind–body cueing was provided. In some studies, foundations were provided concurrently with the general Pilates mat-based program, which alleviated pain intensity and decreased disability in patients with NSLBP [22,28,29,30]. In our experimental group, we added two initial sessions before the Pilates program. To date, few studies have encouraged participants to attend basic introductory lessons. Valenza et al. [31] trained all Pilates group participants in core muscle activation during diaphragmatic breathing, resulting in significant pain improvement and clinical improvement in the Roland Morris Disability Questionnaire. Before the Pilates mat exercise program, Batibay et al. [32] trained participants in key Pilates aspects for one session, with similar results.

The mechanism and specific effects of Pilates exercises on pain management and disability reduction are still debated [33,34]. Some studies suggest that core stabilization exercises like Pilates can influence endorphin levels [35] and involve the endogenous opioid system. Additionally, physical activity is a primary intervention for managing non-specific low back pain (NSLBP), helping to decrease pain levels without a definitive recommendation favoring any specific exercise type over another [36]. This may explain why we found no significant difference between groups, as both received similar interventions, despite the experimental group receiving two extra mind–body cueing sessions. There were no adverse events during the intervention in either group, and adherence was high. Concerning disability scores, one study suggested that to achieve better improvements in pain and functional capabilities, patients with NSLBP need to adhere to a minimum training program of 20 h [37]. During this time, motor control and muscle activity in the lumbopelvic region are stimulated, promoting functional stability and improving functional activities [38]. In our study, one group received 16 sessions and the mind–body cueing group received 18 sessions. Although we did not reach the 20-session threshold, both groups improved their disability scores with no differences between groups. This can be explained by previous studies [19] showing better Roland Morris Disability Questionnaire scores after 12 weeks of training compared to 6 weeks. Future investigations could explore the effect of mind–body cueing over a longer intervention period to determine if it leads to more significant differences between groups. Furthermore, the similarity in results between groups may be attributed to supervision during exercise sessions. Participation in the Pilates sessions was excellent, with the mind–body cueing group attending at an average rate of 94.5%, and the group without cueing maintaining a 92.8% attendance rate. The adherence rates between the two groups did not show significant differences (*p* = 0.432). Additionally, there were no adverse effects reported in either group, indicating that the interventions were well tolerated and safe for the participants.

Kinesiophobia, the fear of movement, significantly contributes to persistent pain and disability in individuals with NSLPB, leading to decreased activity levels and increased perceived disability [39]. Previous studies [22,40] found that Pilates exercises reduced kinesiophobia levels in participants with NSLBP compared to minimal or no intervention. Our study aligns with these results, showing significant reductions in TSK scores in both groups, with a notable difference favoring the mind–body cueing group (*p* < 0.05). This suggests that introductory mind–body cueing sessions may play a crucial role in reducing fear of movement in NSLPB participants. This difference may be attributed to the specific practical information provided to the mind–body cueing group regarding Pilates principles, which also target psychological mechanisms [41]. One study suggested that mind–body interventions like Pilates may share similar mediating pathways with other psychological mechanisms such as pain catastrophizing or kinesiophobia [42], and their reduction may mediate other outcomes like pain intensity and physical function. Adherence to physical activity is a primary objective in NSLBP rehabilitation. Boutevillain et al. [43] found that psychological barriers, particularly kinesiophobia, were the main factors influencing physical activity practice in this population. The effectiveness of NSLBP care is related to an individual’s commitment to an exercise program, promoting consistent long-term effects. According to our results, introductory mind–body sessions were beneficial in reducing this barrier and may increase adherence to a Pilates mat-based program, leading to better long-term self-efficacy scores. Future studies should investigate long-term effects and consider the benefit of adding more introductory sessions or mind–body cueing sessions at the midpoint of the intervention.

The statistical analysis revealed that gender significantly influenced both pain reduction (*p* = 0.021) and kinesiophobia reduction (*p* = 0.043), indicating variations in outcomes between males and females. However, no significant correlations were found between age, height, or weight and the outcomes of pain reduction, disability improvement, or kinesiophobia reduction. Specifically, for pain reduction, the *p*-values for age (*p* = 0.856), height (*p* = 0.484), and weight (*p* = 0.988) indicated no significant impact. For disability improvement, the *p*-values for age (*p* = 0.515), height (*p* = 0.276), and weight (*p* = 0.647) were also not significant. Similarly, for kinesiophobia reduction, the *p*-values for age (*p* = 0.634), height (*p* = 0.531), and weight (*p* = 0.734) showed no significant correlations. For categorical variables, ANOVA results showed that education level, marital status, and occupation did not significantly affect the outcomes. Specifically, for pain reduction, the *p*-values were 0.396 for education, 0.457 for marital status, and 0.250 for occupation. For disability improvement, the *p*-values were 0.091 for gender, 0.282 for education, 0.714 for marital status, and 0.394 for occupation. For kinesiophobia reduction, the *p*-values were 0.657 for education, 0.384 for marital status, and 0.759 for occupation.

The primary limitation of this study is the absence of a follow-up period post-intervention to determine if the observed benefits are sustained over time and if the behavioral trends of the variables remain consistent. Additionally, the study did not include a pure control group receiving no intervention, which was excluded for ethical reasons. The inclusion of such a control group would have allowed for more robust comparisons and clearer attribution of observed effects specifically to the Pilates intervention. Without this long-term data and a non-interventional comparison, it is challenging to fully understand the lasting impact of Pilates on chronic non-specific low back pain and the potential for regression or progression of the condition after the cessation of the intervention.

## 5. Conclusions

This study shows that an 8-week Pilates mat-based intervention is effective for pain management and disability in participants with NSLBP. In addition, the inclusion of specific mind–body cueing has an additional notable impact on reducing movement-related fear. These findings carry significant clinical implications, as they suggest that implementing introductory sessions before the Pilates intervention in participants with kinesiophobia will be beneficial to mediate their pain intensity, improve physical function, and, possibly, play an important role in physical activity adherence.

## Figures and Tables

**Table 1:** Demographic characteristics at baseline.

		PMB Group (*n* = 34)	PA Group (*n* = 34)	*p* Value
Age		37.56 ± 10.08	38.5 ± 07.15	0.53
Gender				0.312
	Male	8 (23.52%)	6 (17.64%)	
	Female	26 (76.48%)	27 (82.36%)	
Height (cm)		170 ± 6.5	171 ± 7.2	0.743
Weight (kg)		66.72 ± 12.58	68.02 ± 9.72	0.436
Education				0.81
	Primary	6 (17.64%)		
	Secondary	8 (23.52%)	14 (41.17%)	
	University	20 (58.82%)	18 (52.94%)	
Marital Status				0.219
	Single	8 (23.52%)	12 (35.26%)	
	Married	14 (41.17%)	12 (35.26%)	
	Divorced	12 (35.29%)	10 (29.41%)	
Occupational status				0.72
	Full-time Worker	24 (70.58%)	25 (73.52%)	
	Part-time worker	5 (14.70%)	5 (14.70%)	
	Unemployed	5 (14.70%)	4 (11.76%)	
Duration of LBP (months)		14.9 ± 8.2	16.2 ± 7.8	0.47

A One-way analysis of variance for continuous variables and χ2 test for categorical variables.

**Table 2:** Intervention program.

Pilates Mat	
1. Warm-ups	12. Rowing 4
2. Single leg stretch	13. Pull straps 1
3. Double leg stretch	14. Pull straps 2
4. Criss cross	15. Swimming
5. Single straight leg	16. Teaser 1
6. Roll up	17. Leg pull back
7. Rolling	18. Leg pull front
8. Side kick: front/back	19. Mermaid
9. Side kick: small circles	20. Rolling down
10. Spine twist	21. Cool down
11. Rowing 3	

**Table 3:** Baseline and posttreatment scores for disability, pain, and fear of movement.

		PMB Group (*n* = 34)	PA Group (*n* = 33)		
Variable		Mean ± SD	Mean ± SD	Mean differences and CI between Groups over Time	*p*-Value
VAS	Pre	5.2 ± 1.9	4.9 ± 2.1	−0.3 (−1.27, 0.67)	0.54
	8 weeks	2.8 ± 1.7	3.2 ± 1.9	0.4 (−0.47, 1.27)	0.372
RMDQ	Pre	8.2 ± 2.9	8.9 ± 2.6	0.7 (−0.63, 2.03)	0.31
	8 weeks	3.1 ± 2.6	4.3 ± 3.0	1.2 (−0.15, 2.55)	0.08
TSK	Pre	42.3 ± 6.5	41.2 ± 8.1	−1.1 (−4.63, 2.43)	0.55
	8 weeks	34.6 ± 7.4	38.3 ± 7.5	3.7 (0.10, 7.30)	0.041

PMB: Pilates Mind–Body with Cueing; PA: Pilates Alone; VAS: Visual Analogue Scale; RMDQ: Roland Morris Disability Questionnaire; TSK: Tampa Scale of Kinesiophobia.

**Table 4:** Correlation analysis and *p*-values between sociodemographic variables and outcome measures of pain reduction, disability improvement, and kinesiophobia reduction.

Variable	Gender	Gender	Age	Age	Height	Height	Weight	Weight	Education	Education	Marital Status	Marital Status	Occupation	Occupation
	(*p*-Value)	(r-Value)	(*p*-Value)	(r-Value)	(*p*-Value)	(r-Value)	(*p*-Value)	(r-Value)	(*p*-Value)	(r-Value)	(*p*-Value)	(r-Value)	(*p*-Value)	(r-Value)
Pain Reduction	0.021	0.35	0.856	0.01	0.484	0.04	0.988	0.00	0.396	0.07	0.457	0.06	0.250	0.09
Disability Improvement	0.091	0.22	0.515	0.05	0.276	0.08	0.647	0.02	0.282	0.08	0.714	0.03	0.394	0.07
Kinesiophobia Reduction	0.043	0.31	0.634	0.03	0.531	0.06	0.734	0.01	0.657	0.04	0.384	0.07	0.759	0.02

## Data Availability

Data are contained within the article.
